# Prediction Model for Hospital-Acquired Pressure Ulcer Development: Retrospective Cohort Study

**DOI:** 10.2196/13785

**Published:** 2019-07-18

**Authors:** Sookyung Hyun, Susan Moffatt-Bruce, Cheryl Cooper, Brenda Hixon, Pacharmon Kaewprag

**Affiliations:** 1 College of Nursing Pusan National University Yangsan-si Republic of Korea; 2 Department of Surgery The Ohio State University Wexner Medical Center Columbus, OH United States; 3 Department of Biomedical Informatics The Ohio State University Wexner Medical Center Columbus, OH United States; 4 Central Quality and Education The Ohio State University Wexner Medical Center Columbus, OH United States; 5 Health System Nursing Education The Ohio State University Wexner Medical Center Columbus, OH United States; 6 Department of Computer Engineering Ramkhamhaeng University Bangkok Thailand

**Keywords:** pressure ulcers, electronic health records, logistic model, critical care

## Abstract

**Background:**

A pressure ulcer is injury to the skin or underlying tissue, caused by pressure, friction, and moisture. Hospital-acquired pressure ulcers (HAPUs) may not only result in additional length of hospital stay and associated care costs but also lead to undesirable patient outcomes. Intensive care unit (ICU) patients show higher risk for HAPU development than general patients. We hypothesize that the care team’s decisions relative to HAPU risk assessment and prevention may be better supported by a data-driven, ICU-specific prediction model.

**Objective:**

The aim of this study was to determine whether multiple logistic regression with ICU-specific predictor variables was suitable for ICU HAPU prediction and to compare the performance of the model with the Braden scale on this specific population.

**Methods:**

We conducted a retrospective cohort study by using the data retrieved from the enterprise data warehouse of an academic medical center. Bivariate analyses were performed to compare the HAPU and non-HAPU groups. Multiple logistic regression was used to develop a prediction model with significant predictor variables from the bivariate analyses. Sensitivity, specificity, positive predictive values, negative predictive values, area under the receiver operating characteristic curve (AUC), and Youden index were used to compare with the Braden scale.

**Results:**

The total number of patient encounters studied was 12,654. The number of patients who developed an HAPU during their ICU stay was 735 (5.81% of the incidence rate). Age, gender, weight, diabetes, vasopressor, isolation, endotracheal tube, ventilator episode, Braden score, and ventilator days were significantly associated with HAPU. The overall accuracy of the model was 91.7%, and the AUC was .737. The sensitivity, specificity, positive predictive value, negative predictive value, and Youden index were .650, .693, .211, 956, and .342, respectively. Male patients were 1.5 times more, patients with diabetes were 1.5 times more, and patients under isolation were 3.1 times more likely to have an HAPU than female patients, patients without diabetes, and patients not under isolation, respectively.

**Conclusions:**

Using an extremely large, electronic health record–derived dataset enabled us to compare characteristics of patients who develop an HAPU during their ICU stay with those who did not, and it also enabled us to develop a prediction model from the empirical data. The model showed acceptable performance compared with the Braden scale. The model may assist with clinicians’ decision on risk assessment, in addition to the Braden scale, as it is not difficult to interpret and apply to clinical practice. This approach may support avoidable reductions in HAPU incidence in intensive care.

## Introduction

A pressure ulcer is injury to the skin or underlying tissue, caused by pressure, friction, and moisture. Hospital-acquired pressure ulcers (HAPUs) may not only result in additional length of hospital stay and associated care costs but also lead to undesirable patient outcomes [1,2]. From the data collected at the national and state levels in the United States, a previous research study reported that the incidence rate of HAPU was 4.46% (2313/51,842). The patients who developed HAPUs during the hospital stay had significantly higher in-hospital mortality and mortality within 30 days after discharge [2]. Patients with HAPUs were less likely to discharge home compared with patients without HAPUs; instead, they were transferred to a skilled nursing facility or intermediate care facility [3]. Intensive care unit (ICU) patients have presented higher incidence of HAPUs than general hospital inpatients [4-6]. A hospital stay relative to HAPU may result in additional cost, up to US $700,000 annually [3]; treatment costs for a Stage 3 pressure ulcer range from US $5900 to $14,840, and those for a Stage 4 pressure ulcer range from US $18,730 to $21,410 [3]. Many risk assessment scales exist [7]. The Braden scale [8] is one of the most widely used risk assessment scales [9]. However, none of the existing scales are largely recommended for ICU patients, as they appear to be less accurate when used for ICU patients. ICU patients may be different from general hospital patients, as ICU patients are more likely to be confined to bed and are often dependent on ventilator support [10]. A number of risk factors have been reported, such as history of vascular disease, mechanical ventilation, dopamine treatment, cardiovascular instability, and length of ICU stay [11-13]. However, these risk factors varied across the studies and the significance of the risk factors, and consequently, their relative importance has not yet been clarified [14].

In our previous research studies, we conducted several experiments with ICU electronic health record (EHR) data in terms of the identification of ICU-specific predictors and prediction modeling methods. We explored supervised machine learning methods with the subsets of our data to determine whether machine learning methods were applicable for ICU HAPU prediction [15,16]. Logistic regression showed best performance over machine learning methods, such as naïve Bayes, decision tree, k-nearest neighbor, random forest, and support vector machine [15]. When we compared the performance of Bayesian network, logistic regression, and the Braden scale, the logistic regression and Bayesian network models showed better area under the receiver operating characteristic (ROC) curve (AUC) than the Braden scale. Although the Bayesian network and logistic regression models showed higher specificities than the Braden scale, they presented lower sensitivities than the Braden scale [16]. This indicated that the Bayesian network and logistic regression models were better for ruling out, but they were not good for ruling in. Logistic regression is an appropriate statistical technique that is widely used to identify significant variables and construct a predictive model. When compared with discriminant analysis, logistic regression is limited to 2 nominal groups for the dependent variable; however, it is similar to multiple regression. In addition, logistic regression is robust when the assumptions of multivariate normality and equal variance are not met [17]. This method is relatively easier to interpret than Bayesian networks, as it is not a black-box model. We hypothesize that the care team’s decisions relative to HAPU risk assessment and prevention may be better supported by a data-driven, ICU-specific prediction model of HAPUs. The aim of this study was to determine whether a multiple logistic regression model with ICU-specific predictor variables was suitable for ICU HAPU prediction modeling and to compare the performance of the model with the Braden scale on this specific population.

## Methods

### Research Design

The research design was a retrospective cohort study by using cumulative EHR data. The data were retrieved from the enterprise data warehouse (EDW) of an academic medical center in central Ohio. The medical center had a commercial system that was used for clinical documentation in all ICUs. The EDW compiled EHR data from the various electronic record systems throughout the medical center, such as administrative system, laboratory system, and computerized patient order entry. EDW maintained the entire ICU patient data that ranged over 3 to 13 years, depending on the specific data source at the time of the data extraction. The data extraction was done by the EDW data manager after the institutional review board had approved the study protocol, and then deidentified data were provided to the research team.

### Dataset

We obtained 4 years of ICU data of the patients who had been admitted to the ICU between January 1, 2007, and December 31, 2010. Details regarding data cleaning and preparation process were provided in another journal publication [16].

In terms of defining the dependent variable, we used an International Classification of Diseases, Ninth Revision (ICD-9), code. An individual patient has a list of discharge diagnoses, and we reviewed the patients’ discharge diagnosis data. If a patient had an ICD-9 code that represented a pressure ulcer on the list of discharge diagnoses, the patient was classified into the HAPU group. If not, the patient was classified into the non-HAPU group. Patients who had a pressure ulcer at the time of ICU admission were excluded. The total number of patient encounters was 12,654. The number of patients who developed an HAPU was 735 (5.81%, 735/12,654), and the rest of the patients did not develop an HAPU during their ICU stay.

Regarding independent variables, we used demographic data and clinical data that were available from the extracted ICU data. The data were age, gender, weight, diabetes, vasopressor, isolation, Braden score, endotracheal tube, ventilator episode, length of ICU stay, and ventilator days. Some data elements (gender, diabetes, vasopressor, isolation, and endotracheal tube) were dichotomous, whereas others (age, weight, Braden score, ventilator episode, length of ICU stay, and ventilator days) were continuous.

### Power

To determine a required minimum sample size for logistic regression, we conducted a power analysis using G*Power version 3.1.9.4. (University of Dusseldorf) To achieve 90% of power to correctly reject the null hypothesis with an alpha of .05, a small effect size (odds ratio 1.2) [18], and 2-tailed test, the sample size of 5731 was considered sufficient. On the basis of the guideline, we decided that we had a sufficient sample for the analysis.

### Data Analysis

**Figure 1:**
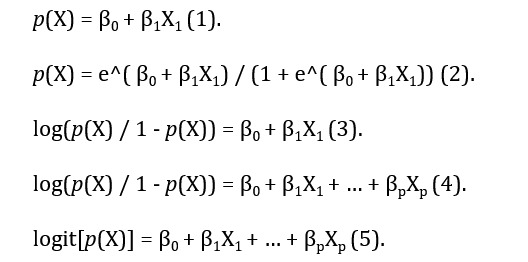
Equations 1-5.

Patient demographics were summarized using descriptive statistics. Comparisons between the HAPU and non-HAPU groups were made using the chi-square test for categorical variables or 2-tailed *t* test for continuous variables. Bivariate analyses were performed to identify predictor variables for ICU HAPU development. Multiple logistic regression was used to develop a prediction model, with significant predictors from the results of the bivariate analyses. A *P* value of less than .05 was considered to indicate statistical significance. The value of dependent variable falls into 1 of 2 categories, with or without an HAPU. Logistic regression models the probability that the value of dependent variable belongs to a particular category. In a linear regression model, these probabilities (*p*) are represented in Figure 1, equation (1).

*X*_1_ represents an independent variable. β_0_ and β_1_ are unknown constants that represent the intercept and slope terms in the linear model. In logistic regression, we use the logistic function, as described in Figure 1, equation (2).

This formula can be represented as described in Figure 1 equation (3).

The quantity p(*X*)/(1-p(*X*)) is called the odds. It can range from 0 to infinite. The formula can be extended with multiple independent variables, as shown in Figure 1 equations (4) and (5).

In this case, X=(X_1_, …, X_p_) are *p* predictors.

For evaluation of model performance, AUC, Youden index, sensitivity, specificity, positive predictive values, and negative predictive values were compared with the Braden scale.

## Results

Table 1 illustrates the comparison of the HAPU and non-HAPU groups. The average age of the HAPU group was 60.5 years and that of the non-HAPU group was 58.4 years. Male patients were 460 (62.6%) of the cases in the HAPU group, compared with 6720 (56.4%) in the non-HAPU group.

Among the patients in the HAPU group, sacrum and buttock were the most common body sites where the HAPUs developed (Table 2).

In the HAPU group, 432 (58.8%) patients had information about their HAPU stages. Stage 2 was most frequent, followed by Stage 4 and Stage 3 (Table 3).

**Table 1:** Comparison of characteristics between the patients with a hospital-acquired pressure ulcer and those without (N=12,654).

Variable		HAPU^a^ (N=735)	Non-HAPU (N=11,919)	*P* value
Age (years), mean (SD)		60.5 (15.5)	58.4 (15.5)	<.001
**Gender, n (%)**				
	Female	275 (37.4)	5199 (43.62)	0.001
	Male	460 (62.6)	6720 (56.38)	—^b^
Weight (lbs), mean (SD)^c^		216.1 (82.7)	200.6 (64.0)	<.001
**Diabetes, n (%)**				
	Present	308 (41.9)	3396 (28.49)	<.001
	Absent	427 (58.1)	8523 (71.51)	—
**Vasopressor, n (%)**				
	Yes	23 (3.1)	238 (2.00)	0.04
	No	712 (96.9)	11,681 (98.00)	—
**Isolation, n (%)**				
	Yes	294 (40.0)	1623 (13.62)	<.001
	No	441 (60.0)	10,296 (86.38)	—
**Endotracheal tube, n (%)**				
	Yes	518 (70.5)	5311 (44.56)	<.001
	No	217 (29.5)	6608 (55.44)	—
**Ventilator episode, n (%)^c^**				
	0	61 (10.5)	1167 (18.01)	<.001
	1	294 (50.8)	3476 (53.66)	—
	2	134 (23.1)	1224 (18.89)	—
	3	46 (7.9)	420 (6.48)	—
	4	27 (4.7)	124 (1.91)	—
	5	10 (1.7)	45 (0.69)	—
	>5	7 (1.3)	22 (0.34)	—
Braden score, mean (SD)^c^		11.9 (2.3)	14.2 (3.6)	<.001
Length of intensive care unit stay (days), mean (SD)		12.6 (13.5)	12.0 (12.5)	.26
Ventilator days, mean (SD)^c^		10.6 (14.4)	5.7 (8.7)	<.001

^a^HAPU: hospital-acquired pressure ulcer.

^b^Not applicable.

^c^The numbers in the columns may not add up to 12,654 because of missing data.

**Table 2:** The body locations of the hospital-acquired pressure ulcers (N=887).

Body location^a^	n (%)
Shoulder blades	9 (1.0)
Elbow	5 (0.6)
Sacrum	509 (57.4)
Hip	39 (4.4)
Buttock	155 (17.5)
Ankle	9 (1.0)
Heel	56 (6.3)
Others	82 (9.2)
Not specified	23 (2.6)

^a^A patient might have multiple body sites of pressure ulcer.

**Table 3:** The categories of hospital-acquired pressure ulcers (N=432).

Stage^a^	n (%)
II	160 (46.8)
III	49 (14.3)
IV	60 (17.5)
Unstageable	26 (7.6)
Not specified	47 (13.7)

^a^Total may not add up to 735 because of missing data.

As a result of the bivariate analyses, age, gender, weight, diabetes, vasopressor, isolation, endotracheal tube, ventilator episode, Braden score, and ventilator days were significantly associated with HAPU presence at an alpha of .05 as a significance level. Length of ICU stay was not found to be significant in our dataset. We conducted a multiple logistic regression analysis using the 10 predictor variables. A test of full model against a constant-only model was statistically significant (X^2^_10_=403.3; *P*<.001). Hosmer-Lemeshow statistic has a significance of 0.323, which means that it is not statistically significant; therefore, the model is a good fit. Nagelkerke *R*^2^ was 0.132. The Wald criterion demonstrated that gender (male; *P*<.001), diabetes (*P*<.001), isolation (*P*<.001), Braden score (*P*<.001), and ventilator days (*P*=.001) made a significant contribution to pressure ulcer prediction (Table 4). The full model tested is shown below:

logit[
*p* (X)] = -.734 + .003*Age + .375*Male + .001*Weight + .397*Diabetes + .181*Vasopressor + 1.130*Isolation -.313*Endotracheal tube + .093*Ventilator episode - .218*Braden score + .014*Ventilator days (6).

**Table 4:** Multiple logistic regression results.

Variable (category)	Beta	SE	*P* value	Odds ratio (95% CI)
Age	.003	0.003	.26	1.003 (0.997-1.009)
Gender (male)	.375	0.095	<.001	1.455 (1.207-1.754)
Weight	.001	0.001	.42	1.001 (0.999-1.002)
Diabetes (yes)	.397	0.098	<.001	1.488 (1.227-1.805)
Vasopressor (yes)	.181	0.239	.45	1.199 (0.750-1.915)
Isolation (yes)	1.130	0.096	<.001	3.094 (2.565-3.733)
Endotracheal tube (yes)	–.313	0.174	.07	0.731 (0.520-1.028)
Ventilator episode	.093	0.050	.06	1.098 (0.996-1.210)
Braden score	–.218	0.022	<.001	0.804 (0.770-0.840)
Ventilator days	.014	0.004	.001	1.014 (1.006-1.022)

**Figure 2:**
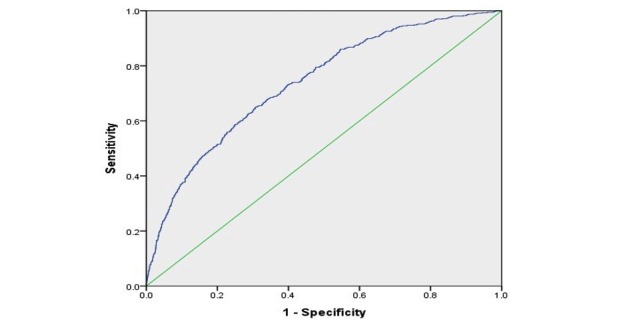
Receiver operating characteristic curve of the prediction model.

**Figure 3:**
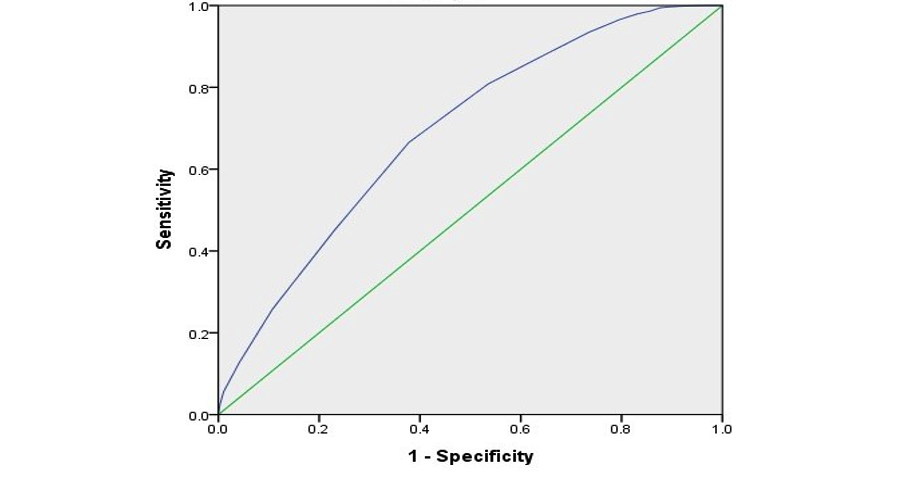
Receiver operating characteristic curve of the Braden scale.

This model had an overall accuracy of 91.7%. The sensitivity, specificity, positive predictive value, and negative predictive value of the Braden scale were 0.665, 0.622, 0.125, and 0.958, and those of the model were 0.650, 0.693, 0.211, and 0.956, respectively. Youden index was 0.287 for the Braden scale and 0.342 for the model. Figure 2 illustrates the ROC curve using the logistic regression model, and the AUC is 0.737 (95% CI 0.727-0.748). Figure 3 shows the ROC curve of the Braden score. The AUC is 0.692 (95% CI 0.682-0.702).

## Discussion

### Principal Findings

We retrieved ICU data of 4 years from an EDW of an academic institution. We conducted a retrospective cohort study to compare demographic and clinical characteristics of the HAPU and non-HAPU groups and identify predictor variables. We performed multiple logistic regression with significant predictor variables to create a prediction model and compared the overall performance of the model to the Braden scale to determine whether a data-driven model was useful to assist clinicians’ decision on ICU HAPU risk assessment and prevention.

A total of 12,654 patients’ demographic and clinical data were used. Among the patients, 735 (5.81%) patients developed HAPUs, whereas 11,919 patients did not develop HAPUs during their ICU stay. In the HAPU group, 432 (58.8%) patients had information about their HAPU stages. According to the national guideline [14], HAPUs are classified into the following categories: Stage 1 means intact skin with nonblanchable redness, Stage 2 is partial thickness skin loss, Stage 3 is full thickness skin loss, Stage 4 indicates full thickness tissue loss, and Unstageable is depth unknown [14]. In the HAPU group, Stage 2 HAPUs were most frequent (46.8%), followed by Stage 4 (17.5%) and Stage 3 (14.3%) HAPUs. It was not clear when Stage 2 progressed to Stage 3 and 4 because of unavailability of data elements. Regarding the body sites of the HAPUs, sacrum (57.4%) was the most frequently reported body location, followed by buttock (17.5%). This finding is consistent with the results of previous studies [6,19], which may relate to the fact that ICU patients are often on the ventilator in a supine position, suggesting a need for attentive care for these body sites. The HAPU and non-HAPU groups were significantly different with respect to age, gender, race/ethnicity, weight, diabetes, vasopressor, isolation, endotracheal tube, ventilator episode, Braden score, and ventilator days. The HAPU group was older, had more male patients, and was heavier than the non-HAPU group. In addition, the HAPU group had significantly longer ventilator days than the non-HAPU group. The HAPU group stayed for longer in the ICU than the non-HAPU group; however, it was interestingly not significant in our data. In terms of risk factors, a number of factors were associated with ICU HAPU, such as history of vascular disease, mechanical ventilation, dopamine treatment, Acute Physiologic Assessment and Chronic Health Evaluation-II score (severity of illness), hypotension, cardiovascular instability, length of ICU stays, and bowel incontinence. However, these factors varied across the studies, and the significance of the factors has not yet been clearly defined [14]. A systematic review reported that age, diabetes, length of ICU stay, vasopressor support, and ventilator days were associated with ICU pressure ulcer development [20]. We included these data elements, in addition to diagnosis and medication dataset, for prediction modeling in this study. Age, gender, weight, diabetes, vasopressor, isolation, endotracheal tube, ventilator episode, Braden score, and ventilator days appeared to be significantly associated with HAPU development in our data. A prediction model was constructed with the significant predictor variables. The overall accuracy of the model was 91.7%, and the AUC of the model was slightly higher than that of the Braden score, indicating the model discriminated the case better than using the Braden score only. It is reported that Youden index is suitable with imbalanced data [21]. The model showed better Youden index than the Braden score. Braden scale showed slightly better sensitivity than the model, although the model showed better positive predictive value than the Braden scale, which indicates the model is slightly better in ruling in patients at risk for HAPU. We explored several machine learning algorithms by using various combination of datasets, such as a dataset with the Braden data only, a dataset with the Braden data plus diagnosis data, a dataset with the Braden data plus medication data, and a dataset with the Braden data plus diagnosis and medication data. We found that the dataset with the Braden data and diagnosis data presented the best predictive validity among the other datasets. In addition, logistic regressions consistently demonstrated better performance than the machine learning algorithms [15]. Next, we examined the applicability of Bayesian networks by using the same datasets and tested the performance of a number of search algorithms, such as greedy hill climbing, repeated hill climbing, Tabu search, and simulated annealing. In the study, we found that the dataset with the Braden data and diagnosis data showed the best average AUC, which was the same result with the previous study. When the predictive validities of the Braden scale, logistic regression, and Bayesian networks were compared, the Bayesian network model and logistic regression showed better AUC than the Braden scale, whereas the Braden scale showed better sensitivity than the Bayesian network model and logistic regression models [16]. The Bayesian network model was not easy to apply to everyday practice, as it was complicated and not easy to interpret. In this research study, we used multiple logistic regression to construct a prediction model with predictor variables that included demographic, diagnosis, medication, and nursing data, and we performed a preliminary evaluation to examine the model performance. The Braden scale is a validated pressure ulcer risk assessment tool, and it is widely used in all kinds of clinical settings [8,9,22]; however, it is not largely recommended, as it showed high false positive rates in ICU patients [12,22]. In evaluation studies on the Braden scale components, only 3 (skin moisture, mobility, and sensory perception) of the components appeared to be significantly associated with pressure ulcer development in ICU patients [13,23]. A systematic review reported that it was not clear whether there was a difference between risk assessment using the Braden scale and risk assessment using clinical judgement in terms of pressure ulcer incidence [7]. The logistic regression model showed better performance than the Braden scale in our data; however, further examination is necessary with a prospective study.

### Limitations

Our data were from 1 single academic institution; thus, the study finding has limited generalizability. Patients who developed an HAPU during their ICU stay were identified by using ICD-9 codes as we were unable to determine what time point the HAPUs actually developed; consequently, ultimate survival analysis was not possible. Stage 1 pressure ulcers were not included into the HAPU group on the basis of the description of the national guideline and the opinion of clinical nursing specialists in our research team; however, it may be suitable to include them into the HAPU group, according to the revised version of the guideline [14]. We used the demographic data and clinical data that were reported significant risk factors for ICU HAPU in the literature as independent variables. Inclusion of more features may result in collinearity issues. Collinearity occurs when there are high correlations among predictor variables, and it may inflate the variances of the parameter estimates. We examined the variance inflation factors of the predictor variables (1.020-1.772), and collinearity could be safely ignored.

### Conclusions

HAPUs are painful and costly complications of hospital care. Using an extremely large, EHR-derived dataset allowed us to compare characteristics of patients who developed an HAPU during their ICU stay with those who did not and to develop a prediction model from the empirical data. The model showed acceptable performance compared with the Braden scale. The model may assist with clinicians’ decision on risk assessment, in addition to the Braden scale, as it is not difficult to interpret and apply to clinical practice. This approach may support avoidable reductions in HAPU incidence in intensive care.

## Abbreviations

AUC: area under the receiver operating characteristic curve
EDW: enterprise data warehouse
EHR: electronic health record
HAPU: hospital-acquired pressure ulcer
ICD-9: International Classification of Diseases, Ninth Revision
ICU: intensive care unit
ROC: receiver operating characteristic
